# Exploration of Ultrasound-Enhanced Transdermal Delivery Efficiency and Anti-Inflammatory Effect of Rutin

**DOI:** 10.3390/ph18040464

**Published:** 2025-03-26

**Authors:** Qing Yue, Bingbing He, Zhenyu Guo, Ningtao Zhang, Mei Zhang, Yufeng Zhang

**Affiliations:** 1 School of Information Science and Engineering, Yunnan University, Kunming 650091, China; yueqing089@163.com; 2 Yunnan Botanee Bio-Technology Group Co., Ltd., Kunming 650106, China; 3 Fundamental Department, Yunnan University Dianchi College, Kunming 650228, China

**Keywords:** rutin, sonophoresis, transdermal drug delivery, anti-inflammatory

## Abstract

**Background**: Rutin is a natural flavonoid extracted primarily from plants with anti-inflammatory and antioxidant properties, and it is highly valuable in the cosmetics industry. However, the poor transdermal permeability of rutin limits its application via transdermal administration. Previous studies have predominantly focused on chemical methods for enhancing penetration. This study investigated the potential of ultrasound as a physical method by which to augment the transdermal absorption and anti-inflammatory effects of rutin. **Method**: Through in vitro diffusion experiments, we analyzed the effects of the ultrasonic frequency and intensity on percutaneous absorption. The optimal ultrasound parameters were determined based on the intradermal retention rate, which is defined as the proportion of intradermal retention to the total penetration. Parameters with higher retention rates were considered optimal. To validate the anti-inflammatory efficacy of rutin delivered using the ultrasound-assisted method, we employed a tape-stripping technique to induce inflammation in BALB/c nude mice. Eight mice were assigned to each treatment group: (A) self-repair (control group), (B) regular rutin treatment, and (C) ultrasound-assisted treatment. **Results**: The research findings indicate that ultrasound frequency and intensity of 1 MHz and 0.2 W/cm^2^, as well as 3 MHz and 0.2 W/cm^2^, result in the maximum proportion of rutin intradermal retention, exhibiting values 1.8 times (using porcine skin) and 2.63 times (using nude mouse skin) higher than those achieved without ultrasound, respectively. Group C showed the shortest recovery time and displayed complete skin barrier function restoration by the fourth day (p<0.05), whereas group A exhibited the slowest recovery. **Conclusions**: This study offers an innovative approach for the transdermal delivery of rutin to facilitate skin barrier function repair.

## 1. Introduction

Rutin, also known as sophorin, vitamin P, or quercetin-3-rutin, is a natural flavonoid found in plants [1,2]. Rutin is a pale-yellow crystalline powder with a molecular formula of C_27_H_30_O_16_ and molecular weight of 610.518 Da. Rutin is nearly insoluble in water but is readily soluble in polar solvents, such as methanol, ethanol, and pyridine [3,4,5]. Because of its anti-inflammatory and antioxidant properties, rutin has been widely used in various types of external cosmetics, including creams, lotions, essences, and masks [6,7,8,9].

Owing to its inherently poor permeability, rutin is insufficient to exert a significant anti-inflammatory effect. Therefore, there is an urgent need to develop strategies that enhance its transdermal delivery. A large number of studies have been conducted on the chemical penetration enhancement of rutin. For example, Das et al. [10], Li et al. [11], and Kajbafvala et al. [12] prepared plant phospholipid rutin complex, rutin-NC gel, and novel rutin transdermal microemulsion to demonstrate the effectiveness of chemical penetration enhancement in enhancing rutin penetration. However, the effectiveness of physical methods in promoting rutin penetration is still unclear.

Common physical techniques include iontophoresis, electroporation, microneedling, and sonophoresis [13,14]. Iontophoresis is generally suitable for molecules that are water-soluble, charged, and smaller than 13 kDa [14,15]. Electroporation involves the application of high-voltage electrical pulses (5–500 V), which can cause cell death, heat-induced drug damage, and protein denaturation [13]. Microneedle therapy is not limited by the molecular weight of the drug or depth of penetration. However, this invasive procedure can damage the integrity of the skin barrier and cause microtrauma and capillary rupture [16]. In contrast, the ultrasonic introduction method is a noninvasive means by which to promote penetration by emitting ultrasonic waves and inducing cavitation to open the skin layer and thereby form water channels to promote penetration. This method enhances the transdermal transport of hydrophilic, lipophilic, and large-molecular-weight compounds, which makes it easier to control the penetration depth of drugs [17,18].

Cavitation is considered to be the primary mechanism by which ultrasound enhances skin permeability. This process occurs when liquids are pulled apart by forces that exceed their tensile strength, thus creating gaps in the stratum corneum that allow drugs to penetrate the skin [19]. Cavitation can be classified as either stable or inertial. Stable cavitation occurs when lower ultrasound intensities induce conservative oscillations in the bubble volume. In contrast, higher ultrasound intensities cause oscillating bubbles to reach their maximum potential size and eventually rupture via a process known as inertial cavitation, which induces physical effects such as shock waves or microjets [20]. During ultrasound delivery, factors such as ultrasound frequency, intensity, duration, and drug characteristics are the main determinants of transdermal penetration efficiency. Low-frequency sonophoresis (LFS, 20 kHz<f<100 kHz) is considered more effective in enhancing penetration, with microjets and shock waves being the primary mechanisms for enhancing skin permeability [21]. Moreover, LFS is more suitable for the penetration of high-molecular-weight compounds and is often used in tissues with deeper target sites [22,23]. In contrast, high-frequency sonophoresis (HFS, >1 MHz) is more effective in increasing the penetration of small-molecular-weight compounds and has a long-term safety profile [24]. Research indicates that increasing the ultrasound intensity and prolonging the exposure time can improve the penetration efficiency; however, the resulting thermal effects must be considered to avoid thermal damage [25]. Therefore, the desired penetration depth can be achieved by appropriately applying ultrasound intensity and frequency to avoid the heat generated by ultrasound introduction [26]. The frequency range of intermediate-frequency sonophoresis (IFS, 100 kHz<f<1 MHz) falls between those of HFS and LFS, thereby providing a compromise for the selection of the penetration frequency [26]. Recently, IFS methods have been used to investigate the effectiveness of transdermal drug delivery.

Several studies have explored ultrasound to enhance transdermal drug delivery in skincare, with HFS being the most studied. Liao et al. [27] used 1 MHz ultrasound at 2 W/cm^2^ to enhance α-arbutin penetration, thus significantly increasing its depth and amount, compared with those of controls, and thereby improving its whitening effect. Maia Filho et al. [28] found that aloe vera gel alone had no significant anti-inflammatory effect on collagenase-induced tendinitis in rats, whereas combining it with 1 MHz ultrasound at 0.5 W/cm^2^ reduced swelling and inflammatory cells. Recently, IFS techniques have been explored to enhance transdermal drug delivery. Park et al. [29] used ultrasound with frequencies of 280 and 350 kHz and an intensity of 0.5 W/cm^2^ for the penetration of nicotinamide and retinol. The results showed that the rates of nicotinamide and retinol enhancement by ultrasound penetration were 402% and 292%, respectively. Yu et al. [26]. reported an ultrasound patch that enhanced the transdermal transport of nicotinamide by inducing mid-frequency (220 kHz) acoustic impedance in the fluid coupling medium between the patch and skin. The experimental results showed that the penetration capacity of nicotinamide increased by 26.2 times after 10 min of ultrasound. In summary, penetration enhancement using IFS and HFS for skincare is effective and safe. However, the anti-inflammatory effects of the ultrasound-enhanced transdermal delivery of rutin, which is a common anti-inflammatory and antioxidant ingredient in skincare products, have not been reported in the literature.

In this study, we investigated the effects of ultrasound-assisted permeation on the enhancement of rutin permeability and the repair of damaged skin barrier function. In the in vitro diffusion experiments, we analyzed the impact of ultrasound frequency and intensity on the percutaneous absorption of pig and mouse skin. Ultrasound parameters were determined based on the cumulative permeation amount per unit area and intradermal retention. To explore the ultrasound-assisted rutin permeation with determined parameters to enhance the anti-inflammatory effects, mice with a damaged skin barrier function using tape stripping were grouped to observe skin barrier function repair via self-repair, regular rutin treatment, and ultrasound-assisted treatment.

## 2. Results

### 2.1. In Vitro Diffusion of Porcine Skin

**Figure 1:**
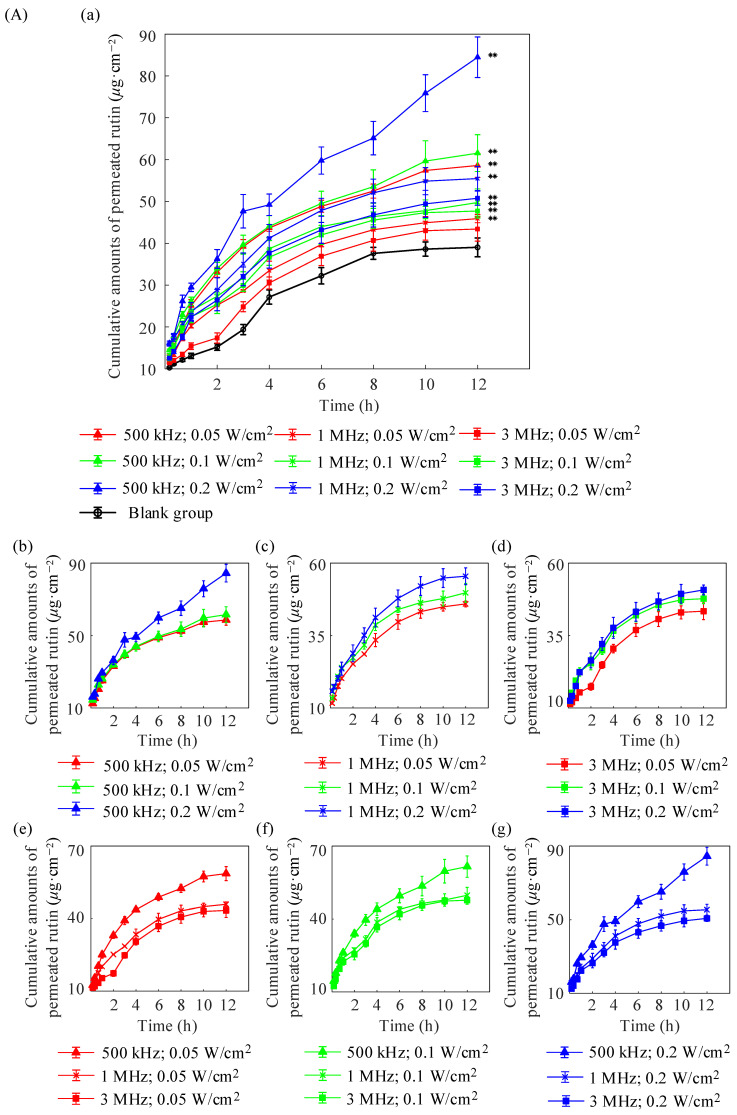

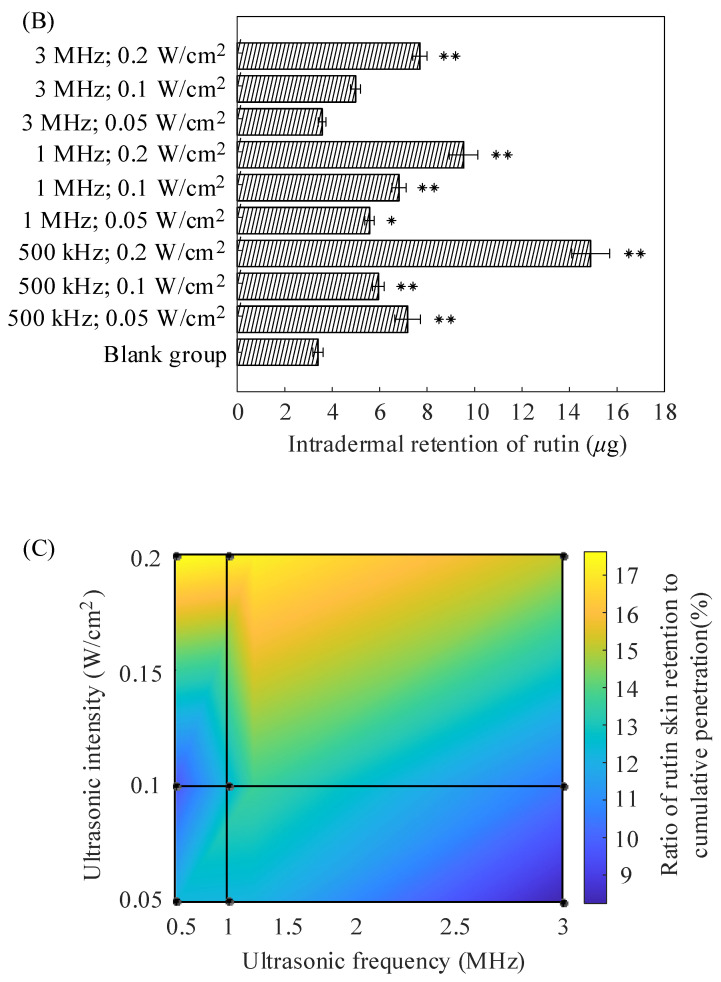
(**A**) Cumulative penetration amount of rutin per unit area in porcine skin with and without ultrasound at different frequencies and intensities (** p<0.01). Panel (a) shows the overall comparison results. Panels (b–d) show the results with different intensities under frequencies of 500 kHz (b), 1 MHz (c), and 3 MHz (d), respectively. Panels (e–g) show the results with different frequencies under intensities of 0.05 W/cm^2^ (e), 0.1 W/cm^2^ (f), and 0.3 W/cm^2^ (g), respectively. Data are presented as means ± standard deviations (N=3). (**B**) Intradermal retention of rutin with and without ultrasound at different frequencies and intensities (* p<0.05; ** p<0.01). Data are presented as means ± standard deviations (N=3). (**C**) Proportion of rutin intradermal retention in the cumulative penetration amount under different ultrasonic frequencies and intensity treatments. (Asterisk symbol indicates a statistically significant difference compared with the blank group).

For the in vitro diffusion experiments, porcine skin samples were divided into a blank control group as well as various treatment groups with different ultrasound frequencies and intensity settings. Three independent replicates were performed for each group (N=3). The cumulative permeation per unit area ($Q_{12h}$), permeation coefficient (P), and steady-state transdermal flux (J) of rutin were calculated after 12 h. Figure 1A shows the comparison curves of the cumulative penetration amount of rutin per unit area in pig skin within 12 h without ultrasound to promote penetration as well as that treated with different ultrasound frequencies and intensities. Figure 1A(a) shows that except for group (3 MHz; 0.1 W/cm^2^), the cumulative penetration amount per unit area of the other groups increased significantly, compared with the group that did not use ultrasound to promote penetration (blank group) (p<0.01). These results are attributable to the reduction in the ultrasound frequency or increase in the ultrasound intensity further enhancing the permeation effect. Compared with the (3 MHz, 0.05 W/cm^2^) group, the other experimental groups had a lower ultrasound frequency or higher ultrasound intensity. The curve for group (500 kHz; 0.2 W/cm^2^) sharply rises, owing to ultrasound thermal effects causing skin burns, which makes the data in this group unreliable. These results are attributable to increases in ultrasound-induced thermal effects causing structural damage to the skin tissue and leading to an abnormal increase in drug permeation. Moreover, the degree of thermal damage varies among samples, which may lead to large variability in the penetration results and thereby affect the consistency and reliability of the data. Figure 1A(b)–(d) show a comparison of the effects of different ultrasonic intensities at the same ultrasonic frequency. The cumulative penetration amount of rutin per unit area increased with an increase in ultrasonic intensity. Figure 1A(e)–(g) show comparisons of different ultrasonic frequencies with the same intensity. The permeability of rutin gradually increases with an increase in the ultrasonic frequency. The curve for a frequency of 500 kHz is always above the curves for frequencies of 1 MHz and 3 MHz, and the 1 MHz frequency result is slightly higher than the 3 MHz frequency result.

Table 1 lists the permeability coefficients calculated according to Equation (2), which directly reflect the speed of permeation. Moreover, the permeability coefficient of group (500 kHz; 0.1 W/cm^2^) is 0.196 ± 0.006 cm/h, which was the highest observed among all groups within the temperature safety range at approximately 1.63 times that of the blank group.

Figure 1B shows a comparison of rutin retention in pig skin in each group after 12 h of in vitro diffusion. The figure shows that rutin retention in the skin increases with an increase in the ultrasound intensity when the ultrasound frequency exceeds 1 MHz. Moreover, group (1 MHz; 0.2 W/cm^2^) exhibits the highest rutin retention among all groups within the given ultrasound frequency range (95% CI: [4.49,7.78],p<0.01), which is approximately 2.86 times that of the blank group. However, there was no significant increase in skin retention for the other two groups, (3 MHz; 0.1 W/cm^2^) and (3 MHz; 0.2 W/cm^2^), compared with the blank group.

Figure 1C illustrates the proportion of rutin skin retention to the total permeation with brighter and darker colors. The horizontal axis represents the ultrasound frequency, and the vertical axis represents the ultrasound intensity. The marked points in the figure indicate the proportion of rutin skin retention corresponding to the ultrasound frequency and intensity used in the experiment. Except for the group with skin burns, (500 kHz; 0.2 W/cm^2^), the (1 MHz; 0.2 W/cm^2^) group exhibited the highest proportion of rutin skin retention, thus providing supportive parameters for the subsequent ultrasound treatments for compromised skin barriers. The color bar on the right represents the corresponding colors for different rutin retention ratios. Higher retention ratios are indicated by bright yellow, and lower ratios are shown in deep blue. The gradient of colors corresponds to varying levels of retention to provide a reference for estimating the permeation effects of ultrasound frequencies and intensities that were not tested in the experiment.

### 2.2. In Vitro Diffusion of Nude Mouse Skin

**Figure 2:**
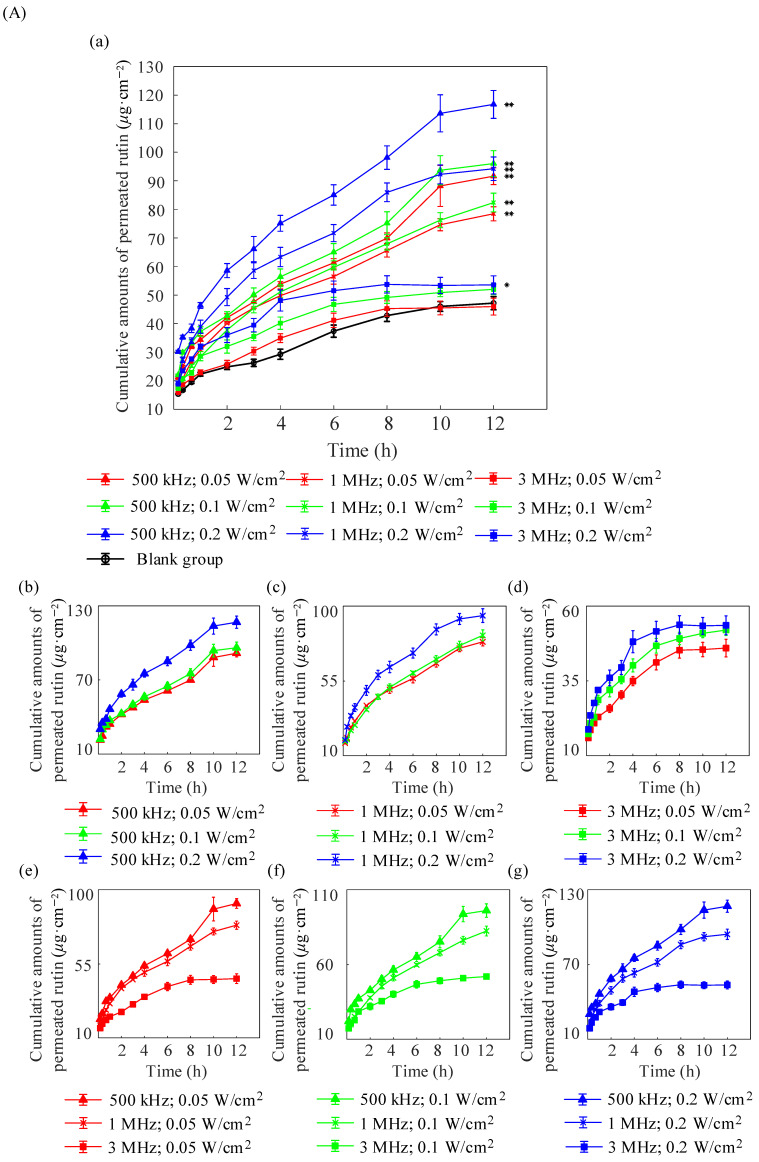

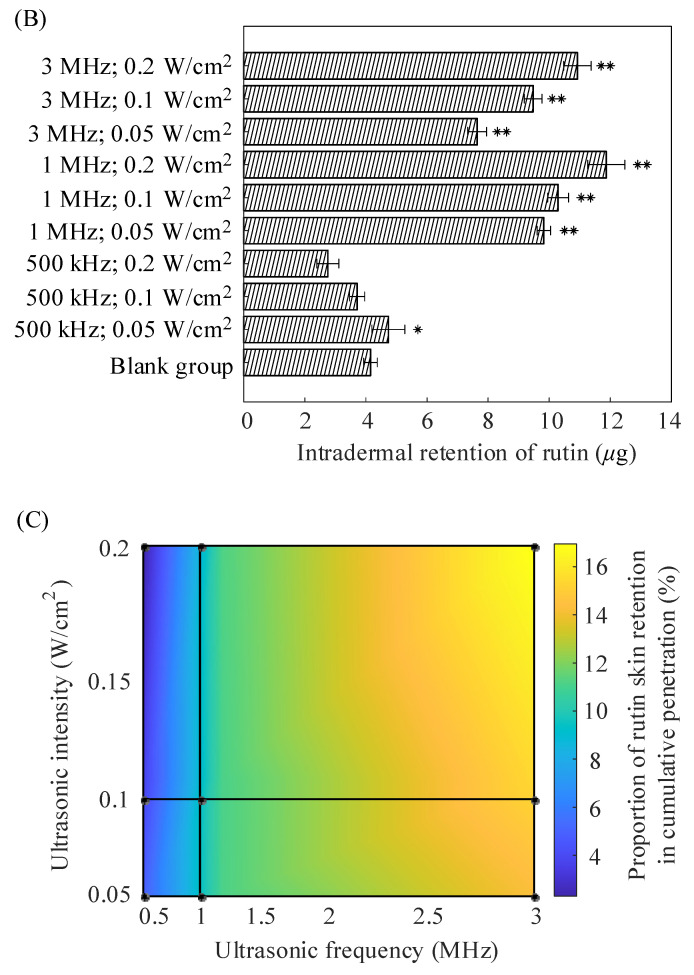
(**A**) Cumulative penetration amounts of rutin per unit area in mouse skin with and without ultrasound at different frequencies and intensities (** p<0.01, * p<0.05). Panel (a) shows the overall comparison results. Panels (b–d) show the results with different intensities under the frequencies 500 kHz (b), 1 MHz (c), and 3 MHz (d), respectively. Figures (e–g) show the results with different frequencies under intensities of 0.05 W/cm^2^ (e), 0.1 W/cm^2^ (f), and 0.3 W/cm^2^ (g), respectively. The data are presented as means ± standard deviations (N=3). (**B**) Intradermal retention of rutin with and without ultrasound at different frequencies and intensities (* p<0.05; ** p<0.01). The data are presented as means ± standard deviations (N=3). (**C**) Proportion of rutin intradermal retention in the cumulative penetration amount under different ultrasonic frequencies and ultrasonic intensity treatments. (Asterisk symbol indicates a statistically significant difference compared with the blank group).

In vitro diffusion experiments were conducted with grouped mouse skin samples to validate the penetration effects of different types of skin. Three repeated tests were performed independently for each group (N=3), and the cumulative permeation amount per unit area ($Q_{12h}$), permeation coefficient (P), and steady-state transdermal flux (J) of rutin were calculated after 12 h. Figure 2A shows comparison curves of the cumulative penetration of rutin per unit area into nude mouse skin under different treatment conditions. With the exception of the (3 MHz; 0.05 W/cm^2^) and (3 MHz; 0.1 W/cm^2^) groups, all other groups showed a significant increase in cumulative rutin penetration per unit area, compared with the blank group (p<0.05). The cumulative permeation amount of rutin at an ultrasound frequency of 3 MHz was significantly lower than those values observed at other frequencies. However, at a frequency of 3 MHz, the permeation amount of nude mouse skin was slightly higher than that of porcine skin samples. Furthermore, for treatments using ultrasound frequencies of 1 MHz and 500 kHz, the cumulative permeation amount per unit area of nude mouse skin was significantly higher than that of porcine skin. The reason for this phenomenon is the thinner back skin of nude mice compared with that of porcine skin, which makes it more prone to permeation. Figure 2A(b)–(d) show comparisons of the permeation results with different ultrasonic intensities at ultrasonic frequencies of 500 kHz, 1 MHz, and 3 MHz, respectively. Figure 2A(e)–(g) show comparisons of the different ultrasonic frequencies at intensities of 0.05 W/cm^2^, 0.1 W/cm^2^, and 0.3 W/cm^2^, respectively. Similar to diffusion in porcine skin, an increase in ultrasound intensity or decrease in ultrasound frequency facilitates rutin permeation.

Table 2 compares the permeability coefficients of rutin in the dorsal skin of nude mice treated using different methods.

Figure 2B shows the amount of rutin retained in the skin of nude mice after 12 h of in vitro diffusion. Except for the (500 kHz; 0.05 W/cm^2^) and (500 kHz; 0.1 W/cm^2^) groups, the skin retention amounts of other groups show significant differences compared with the blank group (p<0.05). Moreover, compared with porcine skin, there was a significant increase in skin retention at ultrasound frequencies of 1 and 3 MHz. Group (1 MHz; 0.2 W/cm^2^) exhibited the highest skin retention ($95\%\;CI:\;\left[6.62,\;8.82\right],\;\;p<0.01$), which was approximately 2.86 times that of the blank group. In contrast to the 1 MHz and 3 MHz frequencies, the skin retention of rutin decreased with an increasing ultrasound intensity at an ultrasound frequency of 500 kHz. These results likely occurred because lower frequencies target deeper layers and increased intensity allows more of the drug to penetrate the receptor chamber, which likely resulted in decreased dermal retention and increased permeation.

Figure 2C shows the proportion of rutin retained in the skin of nude mice, relative to the total penetration amount. Different from pig skin, group (3 MHz; 0.2 W/cm^2^) has the highest proportion. Thus, the corresponding parameter set is the most suitable to select for the subsequent in vivo nude mice experiments.

### 2.3. In Vivo Experiments in Nude Mice

The skin of 24 nude mice was modeled to produce four damage grades: normal skin, mild skin barrier damage, moderate skin barrier damage, and severe skin barrier damage. Each grade was evenly divided and combined into three groups (six mice in each group) for skin repair tests via self-repair (control group), regular rutin treatment, and ultrasound-assisted treatment. After treatment, the skin conditions of the three groups of nude mice improved to different degrees.

**Figure 3:**
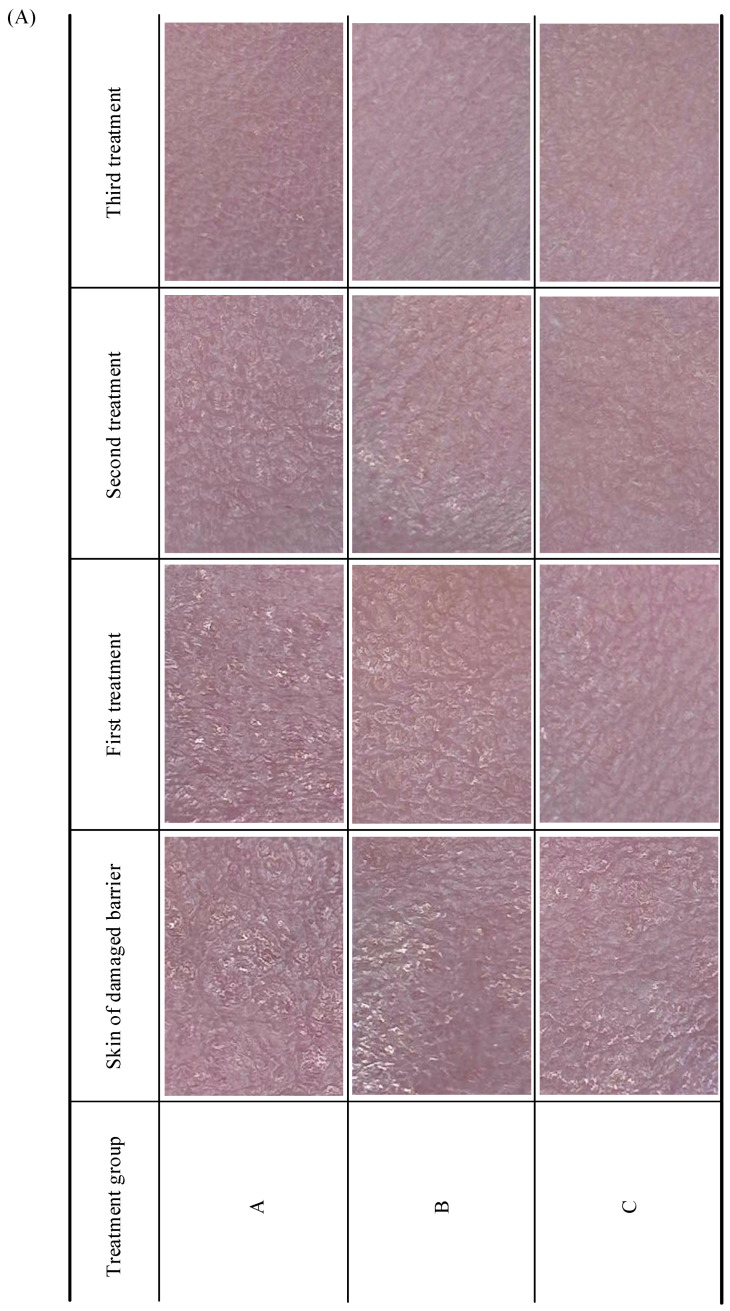

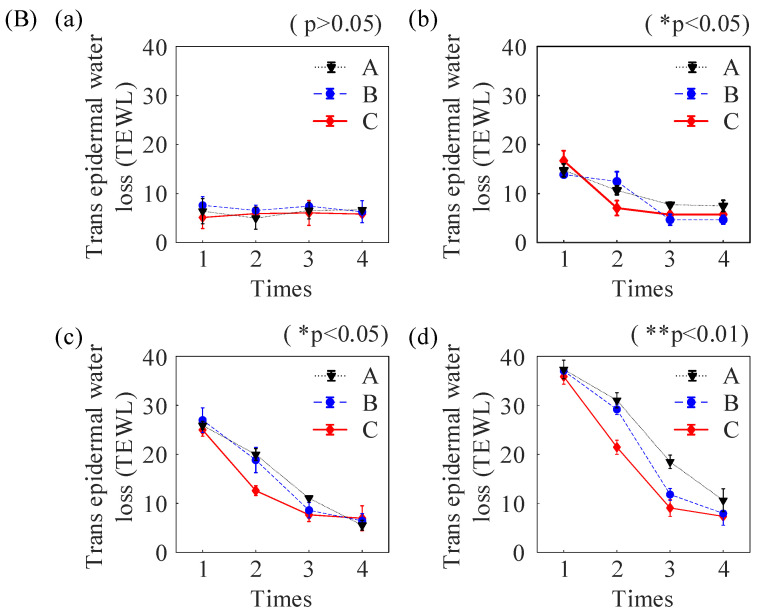
(**A**) Dermoscopy images of nude mice with severe skin barrier damage treated via (A) self-repair (control group), (B) regular rutin treatment, and (C) ultrasound-assisted treatment. Each image magnified 50 times by the DermaLab Combo corresponds to actual skin with a size of 3.4 × 2.5 mm. (**B**) TEWL values measured from the nude mice with normal skin (** p<0.01, * p<0.05) (a) as well as a mildly damaged (b), moderately damaged (c), and severely damaged skin barrier (d) for the self-repair (control group), regular rutin treatment, and ultrasound-assisted treatment groups. In each group, TEWL values were measured in terms of skin barrier damage for the first, second, and third treatments. The data for each treatment are presented as means ± standard deviations (N=10). (Asterisk symbol indicates that there are significant differences in the survival curves among the three groups.).

Figure 3A shows the dermoscopic images of nude mice with severe skin barrier damage treated via self-repair (control group), regular rutin treatment, and ultrasound-assisted treatment. After the third treatment, the skin texture of all nude mice flattened, and the light reflected on the skin surface caused by the damaged barrier nearly disappeared to different degrees. In particular, the skin texture of group C improved significantly after the first treatment, and the light reflection on the skin surface of this group almost disappeared after the second treatment. These results indicate that the time required for skin barrier function repair in group C was the shortest among the three groups.

As the gold standard for assessing skin barrier damage, the TEWL index directly reflects the state of the skin barrier function [30]. To quantitatively evaluate the repair effects of the three treatment groups, Figure 3B shows the TEWL values of the skin measured from the nude mice of groups A (self-repair), B (regular rutin treatment), and C (ultrasound-assisted treatment). In each group, TEWL values were measured at 10 different locations in the treated areas of two mice (N=10) when the skin barrier was damaged: the first, second, and third treatments (Times=4), respectively. For the skin without damage shown in Figure 3B(a), the difference in the measured TEWL values among the three groups was insignificant (p>0.05), indicating that the three treatment methods did not present significantly different effects on normal skin. For the mild, moderate, and severe skin barrier damages shown in Figure 3B(b)–(d), respectively, the TEWL values of group C (ultrasound-assisted treatment) were significantly lower than those observed for groups A (self-repair) and B (regular rutin treatment) (p<0.05). In particular, for the severe damage shown in Figure 3B(d), the TEWL values of group C were significantly lower than those of groups A and B (95% CI: [3.49, 4.02],p<0.01) for all treatments. Notably, the TEWL result in group C is 7.35 ± 0.51 g/(cm^2^·h) for the third treatment, which indicates that the damaged skin barrier was nearly cured after three treatments. Therefore, ultrasound-assisted repair is the most effective treatment for skin damage.

## 3. Discussion

In this study, we investigated the effects of ultrasound frequency and intensity on the ultrasound-assisted penetration of rutin and evaluated the effectiveness of ultrasound-assisted treatment for skin barrier damage. In the in vitro diffusion experiments, isolated porcine skin and isolated mouse skin with different thicknesses were used to find the optimal parameters of 1 MHz and 0.2 W/cm^2^ for porcine skin as well as 3 MHz and 0.2 W/cm^2^ for mouse skin. Based on the in vitro diffusion study, we used the optimal parameters to perform a skin inflammation model experiment on living nude mice. This study illustrated that ultrasound-assisted rutin penetration could restore TEWL values to normal, that is, accelerate the repair of the damaged skin barrier function, more quickly.

To explore the relevant factors affecting the ultrasound-assisted penetration of rutin, we focused on the ultrasonic frequency and intensity. Frequencies of 500 kHz, 1 MHz, and 3 MHz were set in the experiment to evaluate penetration performance. Low-frequency sonophoresis (LFS, 20 kHz<f<100 kHz) has a better drug transdermal delivery effect than high-frequency sonophoresis (HFS, >1 MHz) because the cavitation of LFS on the tissue surface causes a microjet effect that leads to strong tissue penetration and thus, a good therapeutic effect on deep tissues [31,32]. For example, LFS has been used to enhance the transdermal delivery of various drugs such as caffeine, fentanyl, and insulin, with the aim being to penetrate the drug into the bloodstream for transport to the target site [33]. In this study, redness and inflammation caused by skin barrier damage occurred in the epidermal spinous layer, which is relatively shallow under the skin surface [34]. Therefore, the low-frequency range was not considered in this study.

For ultrasound-mediated drug delivery, the ultrasound exposure time influences the efficiency of transdermal drug penetration [25]. In the experiments, the ultrasound exposure time was set to 20 min. In previous studies of ultrasound-mediated drug delivery, the ultrasound exposure time generally ranged from 5 to 20 min [35]. For substances with high molecular weights that are difficult to dissolve in water and lipids, it is necessary to appropriately delay the ultrasound exposure time [36]. Mitragotri et al. [37]. showed that the ultrasound exposure time was positively correlated with the enhancement effect. Zhang investigated the ultrasound-enhanced permeation performance of strychnine using different ultrasound parameters [18]. The results showed that the permeation of strychnine significantly increased within 20 min of ultrasound exposure; however, this increasing trend gradually slowed beyond 20 min. Therefore, we chose an ultrasound exposure time of 20 min for this quantitative study.

It should be noted that the in vitro and in vivo experiments based on isolated porcine and murine skin were conducted to validate the penetration enhancement of rutin. Although it has been demonstrated that ultrasound applications based on optimal parameters can effectively promote rutin permeation without causing skin damage, the safety and efficacy of this method in human skin have not yet been verified. Therefore, further clinical trials in humans are needed to confirm these findings, which will provide a basis for the practical application of this method.

## 4. Materials and Methods

### 4.1. Materials

Rutin was purchased from Shanghai Chenwei Biotechnology Co., Ltd., Shanghai, China. Phosphate-buffered saline (PBS, pH: 7.2–7.4) was acquired from Wuhan Procell Life Technology Co., Ltd., Wuhan, China. NaCl was obtained from Beijing Coolaber Technology Co., Ltd. (Beijing, China). Isoflurane was obtained from Shanghai Yuyan Instrument Co., Ltd., Shanghai, China. All other chemicals and solvents were of analytical grade.

### 4.2. Experimental Instruments

A signal generator (DG1022, RIGOL Technologies, Co., Ltd., Suzhou, China) generated a signal conforming to the resonant frequency of the ultrasonic piezoelectric film (Shenzhen Huajingda Electronic Co., Ltd., Shenzhen, China). The signal was amplified using a power amplifier (ATA-4315, Aigtek Electronic Technology Co., Ltd., Xi’an, China) to actuate the piezoelectric film. By adjusting the amplification factor of the power amplifier, we controlled the ultrasonic intensity generated by the piezoelectric film to the desired level. Additionally, we customized a Franz diffusion cell for in vitro diffusion to accommodate the dimensions of the piezoelectric film.

### 4.3. Skin Sample Preparation

Porcine skin samples were obtained from the skin behind the ears of the pigs at a local slaughterhouse (Yunnan Qiu Muyuan Livestock Slaughterhouse Co., Ltd., Kunming, China) [38]. Specifically, pig ears were collected from the butcher on the same day the pigs were slaughtered at the abattoir. A total of 24 ears were obtained from 3-month-old pigs. After removing the hair from the ears, the skin from the back of the ears was carefully separated, and the subcutaneous fat layer and connective tissue within the dermis were removed to obtain intact skin samples. Thirty BALB/c nude mice were used to collect skin samples. These mice were provided by the Animal Experiment Center of Yunnan University (approval no. YNU20240709). Skin samples were collected from the dorsal skin of the BALB/c nude mice, which were euthanized via cervical dislocation. The skin was carefully peeled off, and the subcutaneous tissue was removed to obtain skin samples. In addition, to minimize the impacts of variations in skin samples on the experimental results, the integrity of each sample was checked. Defective skin samples were discarded, and the thickness of each qualified sample was recorded [39]. After processing, the skin samples were sealed in plastic wrap and bags and then stored at −40 °C. Prior to the experiments, skin samples were thawed and washed using a physiological saline solution. The skin samples were divided into a blank control group and an ultrasound treatment group. The blank control group was subjected to free diffusion without an ultrasound treatment, whereas the ultrasound treatment group was subjected to ultrasound to promote penetration and then divided into 9 groups according to the ultrasound frequency (500 kHz, 1 MHz, and 3 MHz) and ultrasound intensity (0.05 W/cm^2^, 0.1 W/cm^2^, and 0.2 W/cm^2^).

### 4.4. In Vitro Release Studies

Skin penetration experiments were conducted using an improved version of the Franz diffusion cell (vertical). During the experiment, the receptor chamber was filled with 51 mL of PBS solution (pH 7.4), and a magnetic stirring bar was placed in the receptor chamber solution (600 rpm) to ensure uniform mixing. Subsequently, the skin samples were placed between the donor and receptor chambers with the epidermal side facing the donor chamber, and the excess skin on both sides was trimmed to reduce lateral diffusion. The exposed surface area of the skin during diffusion is 9.62 cm^2^. To simulate the human body environment more accurately, the temperature during the experiment was maintained at 37 °C using a circulating water bath in a drug transdermal diffusion apparatus. Subsequently, 5 mL of rutin solution with a concentration of 20 μg/mL was added to the donor chamber. The ultrasound piezoelectric film was placed above the donor chamber to ensure that the surface of the piezoelectric film was in contact with the rutin solution but not submerged. The piezoelectric transducer was driven by a signal generator and power amplifier to produce ultrasound waves with different frequencies and intensities. Next, the ultrasonic treatment was conducted for 20 min. After the ultrasonic treatment, the entrance of the donor chamber was covered with a paraffin film, after which 1 mL of the solution was removed from the receptor chamber every 10 min, 20 min, 40 min, 1 h, 2 h, 3 h, 4 h, 6 h, 8 h, 10 h, and 12 h and then replaced with an equal volume of prewarmed, fresh PBS solution. Each diffusion test was repeated three times, and the collected samples were filtered and directly injected into sample bottles for HPLC analysis. The HPLC analysis results enabled the calculation of the cumulative permeability per unit area and permeability coefficient [40]:

(1) $$Q_{n}=\frac{C_{n}\times V_{0}+\sum_{i=1}^{n-1}C_{i}\times V_{i}}{S},$$

(2) P=J/C,

where $Q_{n}$ (μg/cm^2^) is the cumulative penetration amount of rutin per unit area at the penetration time t=n; $C_{n}$ and $C_{i}$ are the drug mass concentrations measured at the sampling point at t=n and t=i, respectively; $V_{0}$ is the diffusion cell volume 51 mL; $V_{i}$ is the volume of each sampling (1.0 mL); S is the skin diffusion area (1.327 cm^2^); P (cm/h) is the penetration coefficient of rutin into the skin on the back of the ear; J (μg/cm^2^/h) is the steady-state transdermal rate and slope of the curve of cumulative permeability per unit area in 12 h; and C (μg/mL) is the original rutin concentration in the donor chamber.

After completing the 12 h diffusion experiment, the permeation area of the skin samples was removed from the diffusion pool and wiped three times with pure water and ethanol. Subsequently, an absorbent paper was used to remove moisture from the surface of the area. This cleaning step was performed to prevent any residual drugs on the skin surface from affecting the intradermal retention detection. After cleaning, the area was cut into fragments, which were then placed in centrifuge tubes containing 5 mL of methanol. The centrifuge tubes were subjected to ultrasonication for 60 min to ensure the complete dissolution of rutin. Finally, centrifugation was performed at 3000 r/min for 15 min, and the supernatant collected from the centrifuge tubes was filtered into sample bottles for HPLC detection. To ensure that endogenous compounds in the porcine skin did not interfere with the detection of the intradermal retention of rutin, that is, to ensure that the retention times of the endogenous compounds present in the pig skin did not overlap with that of rutin, HPLC detection was conducted on a piece of untreated blank porcine skin. Intradermal retention was calculated using the following formula:

(3) $$A=C_{m}\times V/S,$$

where A (μg) represents the retention amount of rutin in the skin, $C_{m}$ (μg/mL) is the measured mass concentration of the drug remaining in the skin, V is the volume of the added methanol, and S is the skin penetration area.

### 4.5. Animal Treatments

To facilitate the observation of skin barrier damage and recovery, BALB/c nude mice aged 6–8 weeks and weighing 20–25 g were used in the treatment experiments. Throughout the experiment, the nude mice were housed in an environment with controlled temperature and humidity.

The tape-stripping method is a commonly used technique for disrupting the skin barrier function in experiments. This method was first introduced by Wolf in 1939 [41]. The tape-stripping method repeatedly uses tape to peel off the stratum corneum on the surface of the skin, thereby temporarily damaging the skin barrier and causing local skin inflammation. This method has the advantages of simple operation, high repeatability, and no need for external chemical agents.

Tape stripping was used to treat the dorsal skin of the nude mice, and TEWL values served as the standard for evaluating the extent of skin barrier function damage. The nude mice were randomly divided into four groups based on TEWL levels: normal skin (TEWL < 10), mild skin barrier damage (10 ≤ TEWL < 20), moderate skin barrier damage (20 ≤ TEWL < 30), and severe skin barrier damage (30 ≤ TEWL) [42]. Subsequently, the mice were divided equally into three groups (the degree of skin barrier damage in each group was similar) according to the different treatment methods: (A) self-repair (control group), (B) regular rutin treatment, and (C) ultrasound-assisted treatment. The treatments began on the second day after skin barrier disruption and continued every other day until the complete repair of the skin barrier function was achieved in all nude mice. Measurements of skin barrier function indicators, including TEWL and skin dermoscopy images, were scheduled for the second day after each treatment and were taken using TEWL and dermoscopy probes of a skin physiological indicator analyzer (DermaLab Combo, Cortex Technology, Denmark), respectively.

### 4.6. HPLC and Analytical Methods

Quantitative analyses of all samples were conducted using a high-performance liquid chromatography system (Extend-C18 column, 4.6 × 250 mm, 5 μm) with an injection volume of 10 μL, a flow rate of 0.6 mL/min, a detection wavelength of 257 nm, and a column temperature maintained at 30 °C. The initial mobile phase composition consisted of methanol and water in a ratio of 50:50, which was changed to 95:5 after 5 min and restored to the initial ratio after 10 min. This elution method yielded a well-resolved peak for rutin with a retention time of approximate 5.317 min. Within the linear range of 5–100 μg/mL, a standard curve suitable for sample detection was obtained: y=30678.7654x−40.7871 (where y represents the area and x represents the concentration of rutin). A good linear relationship was observed between the concentrations of rutin, with a correlation coefficient of r=0.9984, from which the limit of detection was 1.77 ng/mL. Moreover, a limit of quantity of 5.9 ng/mL was obtained. The sample concentration in the linear range was 20 μg/mL, and the sample was injected continuously 6 times under the above chromatographic conditions, using an injection volume of 10 μL each time. The peak area was measured, and the results showed that the precision of this method was good (RSD = 0.36%).

### 4.7. Statistical Analysis

The results are presented as the means ± standard deviations of at least three experimental datasets. The relevant data were analyzed using analyses of variance (ANOVA) in the SPSS software suite (version 27.0). These analyses were performed to assess differences in cumulative permeation and intradermal retention across groups and were followed by Tukey’s post hoc test to identify which specific groups differed significantly. A Kaplan–Meier analysis was performed to estimate the recovery time of skin barrier function across different treatment groups, and this was followed by the Log-rank test to identify significant differences in recovery times between the groups.

## 5. Conclusions

This study explored the potential of ultrasound as a physical method by which to enhance the transdermal absorption and anti-inflammatory effects of rutin. We analyzed the effects of ultrasound frequency and intensity on transdermal absorption through in vitro diffusion experiments, and determined the optimal parameters based on cumulative permeability per unit area and intradermal retention. Based on the results of an in vitro diffusion study, the optimal parameters were applied to conduct an in vivo nude mouse skin inflammation model experiment. The results demonstrated that the optimal ultrasound permeation parameters are 1 MHz and 0.2 W/cm^2^ for porcine skin as well as 3 MHz and 0.2 W/cm^2^ for the skin of nude mice. Using the latter for barrier repair in nude mice resulted in better performance. In conclusion, this innovative approach provides a mechanism for the transdermal delivery of rutin to facilitate skin barrier function repair.

## Figures and Tables

**Table 1:** Permeability coefficient of rutin for porcine skins subjected to different ultrasonic frequencies and intensity treatments. The $Q_{12h}$ term is the cumulative permeation of rutin per unit area after 12 h of penetration, P (cm/h) is the penetration coefficient of rutin into the skin on the back of the ear, and J (μg/cm^2^/h) is the steady-state transdermal rate and slope of the curve of cumulative permeability per unit area in 12 h. The data are presented as means ± standard deviations (N=3).

Formulations	$Q_{12h}$ (μg/cm^2^)	J (μg/cm^2^/h)	P (cm/h)
Blank group	39.025 ± 1.211	2.396 ± 0.068	0.12 ± 0.003
500 kHz; 0.05 W/cm^2^	58.597 ± 1.892	3.842 ± 0.99	0.192 ± 0.005
500 kHz; 0.1 W/cm^2^	61.542 ± 1.814	3.924 ± 0.109	0.196 ± 0.006
500 kHz; 0.2 W/cm^2^	84.454 ± 1.718	5.695 ± 0.068	0.285 ± 0.004
1 MHz; 0.05 W/cm^2^	45.903 ± 0.963	2.853 ± 0.03	0.143 ± 0.002
1 MHz; 0.1 W/cm^2^	49.738 ± 1.831	3.03 ± 0.111	0.151 ± 0.006
1 MHz; 0.2 W/cm^2^	55.459 ± 1.129	3.307 ± 0.044	0.165 ± 0.002
3 MHz; 0.05 W/cm^2^	43.401 ± 1.737	2.664 ± 0.112	0.133 ± 0.008
3 MHz; 0.1 W/cm^2^	47.714 ± 1.282	2.942 ± 0.065	0.147 ± 0.005
3 MHz; 0.2 W/cm^2^	50.758 ± 1.683	3.185 ± 0.107	0.159 ± 0.006

**Table 2:** Permeability coefficient of rutin under different ultrasonic frequencies and intensity treatments for nude mouse skin. The $Q_{12h}$ term is the cumulative permeation of rutin per unit area after 12 h of penetration, P (cm/h) is the penetration coefficient of rutin into the skin on the back of the ear, and J (μg/cm^2^/h) is the steady-state transdermal rate and slope of the curve of cumulative permeability per unit area over 12 h. The data are expressed as means ± standard deviations (N=3).

Formulations	$Q_{12h}$ (μg/cm^2^)	J (μg/cm^2^/h)	P (cm/h)
Blank group	47.161 ± 1.522	3.93 ± 0.104	0.197 ± 0.004
500 kHz; 0.05 W/cm^2^	91.636 ± 1.714	7.636 ± 0.117	0.382 ± 0.005
500 kHz; 0.1 W/cm^2^	96.056 ± 2.131	8.005 ± 0.145	0.4 ± 0.006
500 kHz; 0.2 W/cm^2^	116.787 ± 2.215	9.732 ± 0.151	0.487 ± 0.006
1 MHz; 0.05 W/cm^2^	78.471 ± 1.568	6.539 ± 0.107	0.327 ± 0.004
1 MHz; 0.1 W/cm^2^	82.411 ± 1.812	6.868 ± 0.123	0.343 ± 0.005
1 MHz; 0.2 W/cm^2^	94.249 ± 2.033	7.854 ± 0.138	0.393 ± 0.006
3 MHz; 0.05 W/cm^2^	45.960 ± 1.719	3.83 ± 0.117	0.192 ± 0.005
3 MHz; 0.1 W/cm^2^	51.987 ± 1.364	4.332 ± 0.093	0.217 ± 0.004
3 MHz; 0.2 W/cm^2^	53.569 ± 1.777	4.464 ± 0.121	0.223 ± 0.005

## Data Availability

The data presented in this study are available on request from the corresponding author. The data are not publicly available due to ethical restrictions related to animal welfare and compliance with institutional animal care and use committee protocols.
